# Evaluation of Safety, Immunogenicity and Efficacy of an Inactivated Bovine Viral Diarrhea Virus (BVDV-1) Vaccine Candidate in Cattle

**DOI:** 10.3390/v18060653

**Published:** 2026-06-08

**Authors:** Semmannan Kalaiyarasu, Niranjan Mishra, Shashi Bhusan Sudhakar, Vijendra Pal Singh, Aniket Sanyal

**Affiliations:** ICAR-National Institute of High Security Animal Diseases, Anand Nagar, Bhopal 462022, Madhya Pradesh, India; kalai_82vetmic@yahoo.co.in (S.K.); sudha.vet@gmail.com (S.B.S.); vijendra61@gmail.com (V.P.S.); aniket.sanyal@gmail.com (A.S.)

**Keywords:** bovine viral diarrhea, inactivated vaccine, cattle, efficacy, immunity

## Abstract

Bovine viral diarrhea (BVD) is a globally significant disease that adversely affects cattle health and productivity, including in India. It is caused by three bovine pestiviruses: *bovine viral diarrhea virus 1* (BVDV-1), BVDV-2, and *HoBi-like pestivirus* (HoBiPeV), which belong to the *Pestivirus* genus within the *Flaviviridae* family. Despite the prevalence of all three pestivirus species in India, no commercial vaccine based on the local circulating strain is currently available. This study evaluates the safety, immunogenicity, and protective efficacy of an inactivated whole-virus BVD vaccine, based on an Indian BVDV-1 strain. The virus was propagated in MDBK cells, inactivated using 3 mM binary ethylenimine (BEI) for 24 h at 37 °C, and formulated with Montanide ISA 61 VG (SEPPIC) in a 50:50 water-in-oil emulsion. Vaccine safety was confirmed in both guinea pigs and bovine calves, with no adverse effects observed. Immunogenicity testing in guinea pigs (*n* = 6) showed neutralizing antibody titres up to 9 log_2_ (1/512). In calves aged 9–12 months (*n* = 3), the vaccine elicited strong humoral and cell-mediated immune responses, with mean neutralizing antibody titres against the homologous BVDV-1 strain reaching 14 log_2_ (1/16,384). Neutralizing antibody levels remained detectable for up to 12 months post vaccination with sustained mean titres of 7 log_2_ (1/128). Notably, titres reported to be adequate for fetal protection (≥9 log_2_ or ≥1/512 were maintained for five months following vaccination. Challenge studies demonstrated complete protection of vaccinated calves against homologous BVDV-1 acute infection. In addition, the vaccine conferred partial cross-protection against heterologous strains including BVDV-2 and HoBiPeV. In a field trial involving 125 cattle, 74% of animals developed protective neutralizing titres (≥7 log_2_ or ≥1/128), while 48% achieved titres reported to be adequate for fetal protection (9 log_2_ or 1/512). Furthermore, 92% of vaccinated cattle maintained neutralizing antibody titres of at least 6 log_2_ (≥1/64) for up to six months post-booster vaccination. A strong positive correlation was observed between guinea pig and bovine antibody responses (R^2^ = 0.6809; *p* < 0.0001), indicating the potential of guinea pigs as a predictive model. Vaccine stability was confirmed for up to 8 months when stored at 4 °C, as demonstrated by the immunogenicity in guinea pigs. Collectively, these findings demonstrate that the locally developed inactivated BVDV-1 vaccine is safe, highly immunogenic, and capable of providing protective immunity against BVDV-1 infection, supporting its potential use in BVD control programs in India.

## 1. Introduction

Bovine viral diarrhea (BVD) is one of the most economically important viral diseases affecting cattle populations globally. BVD is caused by viruses of three bovine pestivirus species, *Pestivirus bovis* (BVDV-1), *Pestivirus tauri* (BVDV-2), and *Pestivirus brazilense* (HoBiPeV), which belong to the *Pestivirus* genus in the *Flaviviridae* family [1]. Infection with bovine pestivirus is known for its complex pathogenesis, wide spectrum of clinical presentations, and its ability to establish persistent infections. The clinical outcomes of BVD range from subclinical to severe, including transient fever, diarrhea, respiratory disease, reproductive failure (including abortion, stillbirths, and congenital defects), and mucosal disease, a fatal condition seen in persistently infected (PI) animals. PI animals result from in utero infection during early gestation and serve as lifelong virus shedders, playing a critical role in the epidemiology of BVDV and sustaining virus circulation within herds, which hamper BVD control efforts [2,3].

Bovine pestiviruses are enveloped with a single-stranded positive-sense RNA genome of about 12.3 kb in length [4], with considerable genetic and antigenic heterogeneity. Thus far, 21 genotypes of BVDV-1 (BVDV-1a to -1u), 4 genotypes of BVDV-2 (BVDV-2a to -2d) and 5 genotypes of HoBiPeV (HoBiPeV-a-e) have been identified [5,6,7]. Hence, vaccination may not prevent all BVDV infections and vaccination failure may occur due to significant antigenic differences between the three bovine pestivirus species (BVDV-1, BVDV-2 and HoBiPeV).

Comprehensive control of BVD requires an integrated approach involving diagnostics, removal of PI animals, biosecurity measures, and immunoprophylaxis. However, vaccination remains a cornerstone of BVD control, aiming to prevent acute infections and fetal transmission. Vaccination against BVD is commonly used to prevent BVDV infection by enhancing immunity in cattle populations with an aim to reduce clinical disease, prevent fetal infection and generate PI calves [3].

Both modified live vaccine (MLV) and killed vaccines are commercially available and have been used in Europe and the Americas to control BVD [8]. Following natural infection or vaccination with a modified live vaccine, the majority of the B cell response (as measured by serum antibodies) is directed against the viral proteins E2 and NS2/3, with minor responses against the E^rns^ and E1 proteins [9], while vaccination with killed vaccines results in serum antibodies directed mainly at the E2 protein [10]. While MLVs tend to elicit a stronger and more rapid immune response, they are associated with safety concerns such as reversion to virulence, fetal infection, post-vaccinal mucosal disease and immunosuppression, particularly in pregnant or stressed animals [11,12,13]. In contrast, inactivated BVD vaccines offer a high safety profile, as they contain inactivated virus particles, eliminating the risk of replication or reversion of virulence. These vaccines are particularly suitable for use in pregnant cows and herds with immunocompromised animals. Hence, inactivated BVD vaccines continue to be widely used by cattle producers due to the safety concerns associated with MLV vaccinations in close time proximity to breeding or during gestation [14]. However, the efficacy of inactivated vaccines depends on several factors, including virus strain selection, adjuvant formulation, and the ability to elicit a strong and lasting immune response.

In India, BVD is widely prevalent in cattle and all three bovine pestivirus species, *Pestivirus bovis* (BVDV-1), *Pestivirus tauri* (BVDV-2), and *Pestivirus brazilense* (HoBiPeV), have been detected, but BVDV-1 occurs predominantly [5,7,15,16,17]. For preventive vaccination against BVD, the development and use of a BVD vaccine having broad coverage against local circulating strains in a country is advocated [3]. Despite the prevalence of BVD, no vaccine based on local circulating strains is currently available, challenging the BVD control efforts in India. Therefore, the goal of this study was to develop an inactivated whole-virus BVD vaccine candidate using an Indian isolate of BVDV-1 and to assess its safety, immunogenicity, and protective efficacy in experimental cattle and to evaluate the safety and immunogenicity in field cattle. The findings of our study demonstrated that the inactivated BVD vaccine developed using a local BVDV-1 strain is safe, immunogenic and effective in protecting cattle against acute challenge-infection with BVDV-1, with reasonable cross-protective immunity against heterologous BVDV-2 and HoBiPeV. The BVD vaccine candidate has the potential for application in the field, offering a promising strategy for BVD control in India to mitigate associated economic losses in the dairy cattle sector.

## 2. Materials and Methods

### 2.1. Virus Strain and Propagation of Seed Virus Stock

Based on the genetic and antigenic characterization data of circulating bovine pestivirus strains in India and the growth kinetics results, a non-cytopathic Indian BVDV-1c isolate (Ind S-10241; GenBank accession numbers—JQ679446 and JQ710873) obtained from the pestivirus repository of ICAR-National Institute of High Security Animal Diseases, Anand Nagar, Bhopal, India, was selected and propagated for 3 additional passages (reaching P-8) on Madin-Darby bovine Kidney (MDBK) cells maintained in Eagle’s minimum essential medium (EMEM) supplemented with 10% horse serum (Gibco, Thermo Fisher Scientific, Waltham, MA, USA), penicillin (100 U/mL), and streptomycin (100 µg/mL). Following infection, the cells were incubated at 37 °C in a 5% CO_2_ atmosphere for 72 h. Virus-containing supernatant was obtained by three cycles of freeze–thaw followed by clarification by low-speed centrifugation (1500× *g* for 10 min) and stored at −80 °C until use. The titre of harvested virus stock was determined by employing an immuno-peroxidase monolayer assay (IPMA) [16] and calculated as 50% tissue culture infective dose (TCID_50_) as previously described [18]. The virus stock was also tested for the presence of other bovine adventitious viruses such as bovine herpes virus (BHV-1), blue tongue virus (BTV), Bovine Parvovirus (BPV), bovine respiratory syncytial virus (BRSV), and bovine adenovirus (BAdV) by PCR/RT-PCR. Virus stock was also tested for the presence of any bacteria, mycoplasma and protozoa employing standard methods.

### 2.2. Inactivation of BVDV-1 Virus

The virus suspension, with a titre of 10^6.0^ TCID_50_/mL, was inactivated using 2-bromoethylamine hydrobromide (BEI) (Sigma-Aldrich, St. Louis, MO, USA) under varying conditions of temperature and time. BEI was freshly prepared prior to use by dissolving in 0.2 N NaOH and incubating at 37 °C for 1 h with continuous mixing to allow activation. BEI was then added to the virus suspension at final concentrations of 0.1 mM, 1 mM, 2 mM, 3 mM, 4 mM, and 5 mM. The mixtures were incubated with gentle agitation at three different temperatures like 4 °C, room temperature (~24 °C), and 37 °C and sampled at 16 h, 24 h, 36 h, and 48 h post-incubation to assess inactivation efficacy. To neutralize residual BEI, 10 µL of sterile 1 M sodium thiosulfate was added per ml of virus suspension, followed by incubation at room temperature for 2 h. Complete inactivation of the virus was confirmed by three consecutive blind passages on MDBK cells followed by virus detection using BVDV-specific antibody-based immuno-peroxidase monolayer assay (IPMA).

### 2.3. Vaccine Formulation

The BVDV-1 virus stock was bulk propagated on MDBK cells using 75cm^2^ TC flasks. The titre of the vaccine virus stock before inactivation was determined and the inactivated virus antigen concentration was quantified by BVDV E^rns^ antigen ELISA (IDEXX, Westbrook, ME, USA). The antigen was emulsified with an oil-based adjuvant, Montanide ISA 61 VG (Kind gift from SEPPIC, Paris, France) in a 50:50 water-in-oil ratio, using high-speed homogenization to ensure uniform emulsification to obtain a stable water-in-oil emulsion.

### 2.4. Animals, Vaccination, Challenge Infection and Sampling

All animal experimental procedures used in this study were reviewed and approved by the ICAR-National Institute of High Security Animal Diseases Institutional Animal Ethics Committee (Reg. No. 234/GO/ReBi-S/Re-L/2000/CCSEA) and Committee for Control and Supervision of Experiments on Animals (CCSEA Approval No. V-11011 (13)/19/2021-CPCSEA-DADF), Government of India. Safe disposal of infected materials and wastes was carried out in accordance with the biosafety norms. At the end of the experiment, as per the CCSEA approved research protocol, all 12 cattle were rehabilitated/adopted as per the Guidelines of CCSEA for Reuse and Rehabilitation of large animals.

### 2.5. Evaluation of Safety and Immunogenicity in Guinea Pigs

**Safety:** Six Dunkin–Hartley guinea pigs (*n* = 6), each weighing between 300 and 400 g, were immunized intramuscularly with 2.0 mL of the test vaccine dose for cattle (double dose of 1/5th of cattle dose) and monitored daily for 15 days.

**Immunogenicity:** Six guinea pigs (*n* = 6) were immunized intramuscularly in gluteus muscle with 1.0 mL of the test vaccine (equivalent to 1/5th of the cattle dose) on day 0, followed by a booster dose on day 21. Three additional guinea pigs served as sham-vaccinated controls (*n* = 3), receiving a placebo treatment (PBS and adjuvant). Prior to immunization, all animals were tested using the virus neutralization test (VNT) and confirmed to be free of BVDV-specific antibodies. Blood samples were collected from all animals on day 0 and subsequently at weekly intervals for up to three months post-booster. Sera were separated and heat-inactivated at 56 °C for 45 min before use in the neutralization assay. The VNT was performed as described in the manuscript elsewhere against BVDV-1. At the end of the experiment, all animals were euthanized in accordance with institutional ethical guidelines.

### 2.6. Evaluation of Safety and Immunogenicity in Cattle Calves

In this study, 12 healthy cattle calves (Malvi breed) aged 9–12 months of either sex were sourced from the Government Cattle Breeding Farm, Agar Malwa, Madhya Pradesh, and used. The animals were housed at the Institutional large animal facility with appropriate veterinary care under standard management conditions and biosafety protocols. Prior to experimentation, all the calves were confirmed negative for bovine pestivirus antibody and antigen by virus neutralization test [16] and real-time RT-PCR [19] respectively. Additionally, these calves were tested free of other co-infecting bovine viruses, such as BHV-1, BTV, BPV, BRSV, BAdV and LSDV by PCR/RT-PCR.

The calves were randomly allocated into four groups, vaccine safety (*n* = 3) (G1), vaccinated-challenged (*n* = 3) (G2), vaccinated-unchallenged (*n* = 3) (G3) and control-challenged (*n* = 3) (G4) groups, and housed in separate animal rooms. The vaccine safety was assessed by administering a double dose (10 mL) to G1 calves and observing for any adverse reactions up to 14 days. The G1 calves were inoculated again with a double dose (10 mL) on day 28 and observed for 7 days to assess the vaccine safety following multiple inoculations. For vaccine immunogenicity and efficacy studies, G2 (*n* = 3) and G3 (*n* = 3) calves were vaccinated intramuscularly in the trapezius muscle with a 5 mL dose followed by a booster dose on day 28, while healthy control calves (G4) were inoculated with placebo injections (containing adjuvant alone) on day 0 and 28. The G2 and G4 calves were subsequently challenged with 5 mL of homologous live BVDV-1 virus (10^6.0^ TCID_50_ per ml, at passage level 8) through the intranasal route at 28 days post-booster (56th day of primary dose). The G3 calves were monitored for 12 months to estimate the level and duration of vaccine immunity response (neutralizing antibody titre).

Blood samples were collected from all animals via the jugular vein, both with and without EDTA, on days 0, 1, 3, and 7 post-immunization, as well as on day 0 (prior to challenge), daily from days 1 to 14 post-challenge, on days 21 and 28, and then at weekly intervals up to 6 months, and subsequently at monthly intervals up to 12 months. Samples collected with EDTA were used immediately for the isolation of peripheral blood mononuclear cells (PBMC) using Histopaque 1077 (Sigma, USA), and aliquots were stored in TRIreagent (Sigma-Aldrich, USA) at −80 °C for further use. Nasal swabs in VTM were collected on day 0 (prior to immunization), on days 1, 2, and 3 post-immunization, as well as on day 0 (pre-challenge) and daily up to 14 days post-challenge (dpc). In addition to the weekly collection of blood and serum from the G3 group of calves, nasal swabs were also collected intermittently throughout the study to confirm their BVDV-free status.

Peripheral blood leukocytes (PBLs) were isolated from EDTA-anticoagulated blood by density gradient centrifugation using Histopaque-1077 (Sigma, USA) at 1000 rpm for 40 min, following a previously described protocol [20], with a modification involving red blood cell lysis using 0.8% ammonium chloride. In parallel, blood samples collected without anticoagulant were allowed to clot at room temperature, and serum was obtained by centrifugation at 5000 rpm for 5 min. All PBL and serum samples were aliquoted and stored at −80 °C until further use.

### 2.7. Clinical Observation of Calves Following Vaccination and Challenge

After vaccination as well as challenge infection, all calves were monitored daily for 14 days to assess clinical responses and any adverse reactions. Observations included general demeanor, rectal temperature, respiratory rate, feed intake, nasal discharge, ocular discharge, salivation, and signs of diarrhea. Injection sites were examined for local swelling, redness, or pain. Body temperature was measured using a digital rectal thermometer, and any animal exhibiting a temperature above 104 °F was considered febrile. All clinical signs were recorded and scored using a standardized system described by [21]. Variation in the clinical signs (respiratory signs, nasal discharge, ocular discharge, depression, and diarrhea) was assigned a numeric score from 0 to 4 (0 = normal, 1 = slight, 2 = mild, 3 = moderate, 4 = severe). A composite clinical score was calculated daily for each animal by summing the scores of these five variables.

### 2.8. Detection of Neutralizing Antibodies Against BVDV-1, BVDV-2 and HoBiPeV

BVDV-1 neutralizing antibodies in serum samples were detected by virus neutralization test (VNT) following the previously described procedure in 96 well plate [16]. Briefly, serial two-fold (starting dilution 1/4) heat-inactivated (56 °C for 45 min) serum samples (50 µL) were mixed with 50 µL of 200 TCID_50_ virus (BVDV-1 isolate Ind S-10241) and incubated for 1 h at 37 °C with 5% CO_2_. Then, the inoculum was replaced with 100 µL of maintenance medium (Eagle’s minimum essential medium, Sigma, USA) with 5% horse serum. Following 72 h incubation, the plates were subjected to an immuno-peroxidase monolayer assay (IPMA) as previously described [16]. Neutralizing antibody titres were expressed as log 2 of the reciprocal of the highest dilution completely neutralizing 200 TCID_50_ virus. The neutralizing antibody titre of 3 log_2_ (1/8) or above was considered positive and as an indicator of seroconversion [3]. BVDV-1c positive serum samples were further subjected to a cross-neutralization test to determine the neutralizing antibody titre against BVDV-1b (isolate Ind S-1449), BVDV-2 (BVDV-2 isolate Ind-141353) and HoBiPeV isolate (HoBIPeV isolate IndBHA5309/12) as described previously [5].

### 2.9. Assessment of Virus Shedding in BVDV-1 Challenged Animals

The control-challenged and vaccinated-challenged calves were assessed for BVDV-1 shedding by real-time RT-PCR and virus isolation for a period of three weeks following the challenge infection.

**(a)**  **Real time RT-PCR:**

Viral RNA was extracted from 140 μL of each nasal swab sample using the QIAamp Viral RNA Mini Kit (Qiagen, Hilden, Germany), while the viral RNA from peripheral blood leukocytes (PBLs) was extracted using the RNeasy Mini Kit (Qiagen, Germany) in accordance with the manufacturer’s instructions and stored at −80 °C until use. To assess the presence of the BVDV genome, RNA extracted from the nasal swabs and PBL samples was tested by a BVDV-1-specific TaqMan real-time RT-PCR [22] on a Bio Rad CFX96 real-time PCR machine (BioRad, Hercules, CA, USA), using the SuperScript III Platinum One-Step quantitative RT-PCR kit (Invitrogen, Carlsbad, CA, USA).

**(b)**  **Virus isolation:**

Peripheral blood leukocyte (PBL) and nasal swab samples were subjected to virus isolation as described previously [16]. Briefly, samples were inoculated onto sub-confluent monolayers of MDBK cells cultured in 6-well plates and incubated at 37 °C for 1 h. Following adsorption, the inoculum was replaced with maintenance medium containing 5% horse serum, and the cultures were incubated for 72 h. Subsequently, cells were subjected to three freeze–thaw cycles, and the clarified supernatants were subjected to the next passage in 96-well plates and analyzed by immuno-peroxidase monolayer assay (IPMA) using pan-pestivirus and BVDV-1-specific monoclonal antibodies (mAbs) obtained from the Veterinary Laboratory Agency (Weybridge, UK), as previously described [16].

### 2.10. Evaluation of IFN-Gamma Response

Serum concentration of IFN-gamma was measured from day 0 to 56 (day of challenge) as an indicator of cell-mediated immune response. The assay was performed using a commercial ELISA kit (CUSABIO, Wuhan, China) according to the manufacturer’s instructions. IFN-γ concentrations were calculated from the standard curve generated with the kit standards.

### 2.11. Assessment of Vaccine Safety and Immunogenicity in Cattle Under Field Conditions

The safety and immunogenicity of the inactivated BVDV-1 vaccine were also evaluated under field settings in a total of 125 cattle (crossbred and indigenous breeds of both sexes) housed at a cattle shelter home (Mahamrutanjay Gau Sewa Sadan, Kokta, Bhopal, Madhya Pradesh) following pre-screening of the animals for health and BVDV antibody status. Of the total of 125 cattle under field trial, 100 animals (*n* = 100) received a 5 mL intramuscular dose of the vaccine on day 0 (primary vaccination) and a booster dose on day 28, whereas another 25 animals (*n* = 25) received placebo injections containing adjuvant alone on day 0 and 28. To assess the humoral immune response to vaccination, virus neutralization (VN) antibody titres were measured by VNT at multiple time points up to 6 months post-booster vaccination (day 0, 30, 60, 90, 140 and 200 DPV). The serum samples collected on 90 DPV were subjected to a cross-neutralization test to determine the neutralizing antibody titres against BVDV-2 (BVDV-2 isolate Ind-141353) and HoBiPeV (HoBIPeV isolate IndBHA5309/12) as previously described [5].

### 2.12. Evaluation of Vaccine Stability

An emulsion (50 mL) of vaccine was prepared and stored at 4 °C for a period of up to 8 months. To assess its stability, three guinea pigs were immunized with a primary and a booster dose on day 0 and day 21 respectively, and their antibody titres were evaluated periodically over a three-month period by virus neutralization test.

### 2.13. Statistical Analysis

All data were analyzed using GraphPad Prism software 8.0.2 (GraphPad Software, San Diego, CA, USA). Antibody titres were expressed as mean ± standard deviation (SD). Comparisons between groups were performed using analysis of variance (ANOVA), and a *p*-value < 0.05 was considered statistically significant.

## 3. Results

### 3.1. Identity of Vaccine Virus

The identity of the BVDV-1 vaccine candidate virus strain (BVDV-1c isolate Ind S-10241, GenBank Acc. No JQ679446 and JQ710873) was confirmed by genomic sequence analysis results.

### 3.2. Preparation of Vaccine

The MDBK cells used for the culture and propagation of BVDV-1 isolate were tested free from extraneous agents, including bacteria, fungi, and mycoplasma, bovine pestiviruses (BVDV-1, BVDV-2, HoBiPeV) and other adventitious bovine viruses (BHV-1, BTV, BPV, BRSV, and BAdV). The selected BVDV-1 isolate (BVDV-1c isolate Ind S-10241) achieved a maximum titre of 2 × 10^6.5^ TCID_50_/mL at an MOI of 0.1, as revealed by a growth kinetics study. A virus inactivation kinetics study revealed that treatment with 3 mM BEI for 24 h at 37 °C was optimal for complete and reliable inactivation of the BVDV-1 virus with no detectable residual infectivity across multiple blind passages on MDBK cells. The vaccine virus was emulsified with adjuvant Montanide ISA 61 VG (SEPPIC, France) in a 50:50 water-in-oil ratio and was used in the experimental trials.

### 3.3. Safety and Immunogenicity in Guinea Pigs

**Figure 1:**
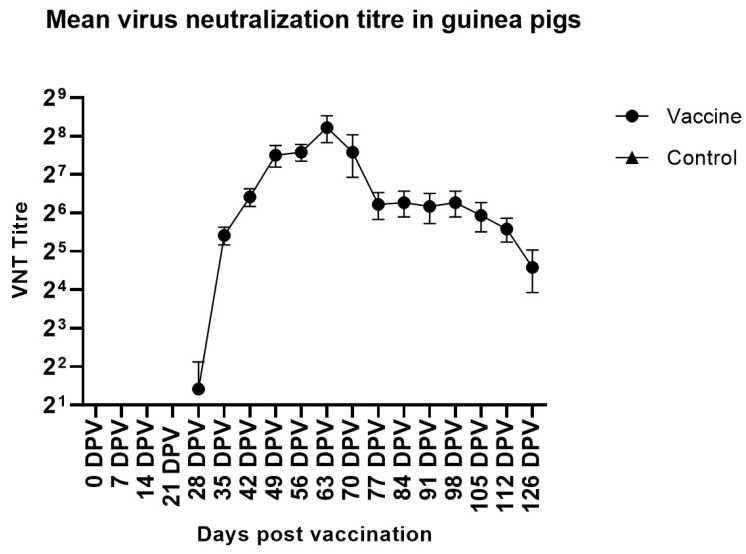
Kinetics of serum immune response (VNT titres in log_2_ units) to inactivated BVDV-1 vaccine in guinea pigs. Control animals had negative (zero) VNT titres and were therefore not included on the logarithmic axis.

No local or systemic adverse reactions were observed in any of the guinea pigs immunized with a double dose of immunogen. All six guinea pigs immunized with the candidate vaccine developed a detectable neutralizing antibody response against BVDV-1 following vaccination. No neutralizing antibodies were detected in any animals at day 0, confirming the absence of pre-existing BVDV-specific immunity. A low level of neutralizing antibodies, 3 log_2_ (titre of 1/8), was first detected in 2 out of 6 animals by day 28 post-vaccination. A marked increase in antibody titres was observed after day 35, with all vaccinated animals demonstrating robust neutralizing responses. The mean antibody titre at this point was 5.41 log_2_ (1/42.67), ranging from 5 to 6 log_2_ (1/32–1/64). Peak neutralizing antibody levels were recorded between day 63 and day 70, with titres ranging from 6 to 9 log_2_ (1/64 to 1/512) (Figure 1). Following the peak response, antibody titres gradually plateaued and remained relatively stable until day 105 post-vaccination. By the end of the observation period (day 126), a decline in antibody levels was noted, with a mean titre of 4.6 log_2_ (1/24) (range: 1/8 to 1/64). In contrast, none of the sham-vaccinated control animals (*n* = 3) developed detectable neutralizing antibodies throughout the study, confirming the specificity of the immune response elicited by the candidate vaccine. No adverse reactions or clinical signs were observed in any of the animals during the study. All animals remained healthy until the end of the experiment.

### 3.4. Vaccine Safety in Experimental Cattle

No local or systemic adverse reactions were observed in experimental cattle calves during 14 days of observation period in the vaccine safety group (G1). Similarly, no significant adverse effects or clinical abnormalities were observed in any of the vaccinated calves (G2 and G3) during the observation period. Mild and transient elevation in rectal temperature was evident only in a few animals within 24 h post-vaccination, which resolved without intervention.

### 3.5. Antibody Responses Following Vaccination and Challenge Infection (Experimental Cattle)

**Figure 2:**
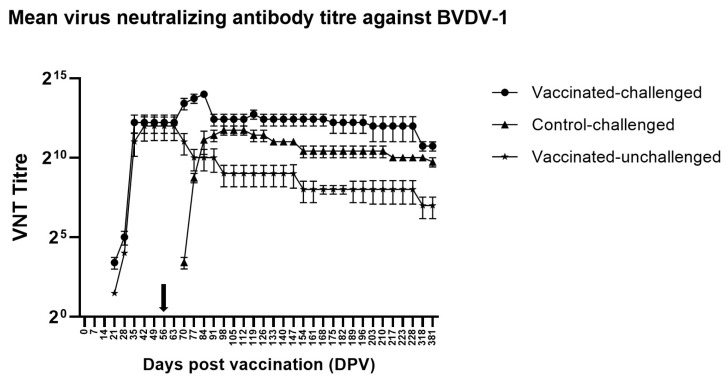
Kinetics of serum immune response (VNT titres in log_2_ units) of cattle calves immunized with inactivated BVDV-1 vaccine followed by challenge with live BVDV-1. Challenge time point (56 dpv) is indicated by an arrow mark.

The virus neutralization test (VNT) was used to evaluate the protective antibody response up to one year following primary vaccination. No virus-neutralizing antibodies were detected in the sham-vaccinated control group (G4) prior to challenge. In contrast, vaccinated calves developed detectable neutralizing antibodies by 21 days post-vaccination (DPV), with two out of six animals (G2 and G3) exhibiting titres of 4 log_2_ (1/16) (Figure 2). By 28 DPV, all vaccinated calves had detectable neutralizing antibody titres ranging from 3 to 6 log_2_ (1/8 to 1/64). On the day of challenge (56 DPV), the mean neutralizing antibody titre in the vaccinated group reached 12.2 log_2_ (1/4778.7), with individual titres ranging from 11 to 13 log_2_ (1/2048 to 1/8192) (Figure 2). Following the challenge, VN titres increased further, peaking at 14 log_2_ (1/16,384) on 84 DPV (four weeks post-challenge). Thereafter, antibody levels gradually declined but remained robust, with a mean titre of 10.73 log_2_ (1/1706.6) maintained through the end of the study at 381 DPV.

In the control-challenged group (G4), neutralizing antibodies began to appear by 14 days post-challenge (DPC), with a mean titre of 3.4 log_2_ (1/10.7). Antibody titres continued to rise, reaching a peak mean titre of 11.7 log_2_ (1/3413.3) by 98 DPC, followed by a gradual decline to 9.7 log_2_ (1/853.3) at the end of study period.

The duration of the immune response following vaccination was assessed in the vaccinated but unchallenged experimental cattle group (G3). The results showed that the neutralizing antibody titres followed a similar pattern of G2 up to 63 DPV, reaching a peak of 12 log_2_ (Figure 2). Thereafter, titres declined gradually over time. Protective levels of neutralizing antibodies against BVDV-1, defined as ≥7 log_2_ (≥1/128), were maintained for up to 12 months post-vaccination (Figure 2). Specifically, a mean titre of 9 log_2_ (1/512) was sustained for five months, 8 log_2_ (1/256) for nine months, and 7 log_2_ (1/128) for 12 months post-vaccination. The antibody titre reported to be adequate for fetal protection (≥9 log_2_ or ≥1/512) persisted for five months following immunization (Figure 2).

**Figure 3:**
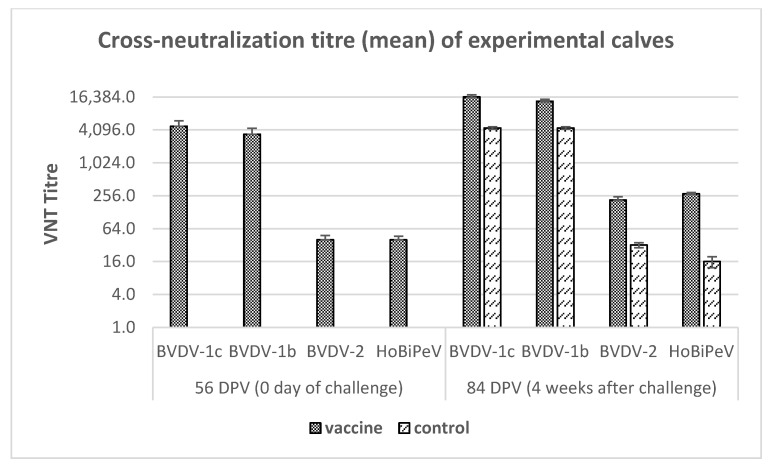
Mean cross-neutralization titre of sera from inactivated BVDV-1 vaccinated calves against heterologous pestivirus strains (BVDV-1b, BVDV-2 and HoBiPeV), before and after challenge infection (BVDV-1c virus).

Cross-neutralization of heterologous bovine pestiviruses with serum from calves at 56 DPV (0 day of challenge infection) and 84 DPV (four weeks after challenge infection) revealed equivalent titre against BVDV-1b and BVDV-1c strains, while there was a 7–8 (56 DPV) and 6–7 (84 DPV) fold reduction in titre against BVDV-2 and HoBiPeV (Figure 3), indicating a strong humoral immunity against homologous virus (BVDV-1) and a moderate level of cross-protective immunity.

### 3.6. Vaccine Efficacy in Experimental Cattle

#### 3.6.1. Clinical Observations

**Figure 4:**
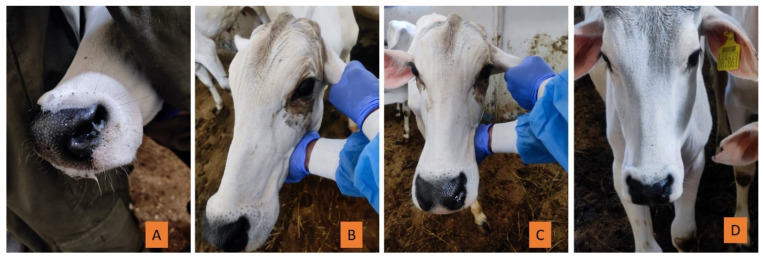
Clinical signs observed in calves following BVDV-1 challenge infection. (**A**) Nasal discharge and (**B**,**C**) ocular discharge observed in control-challenged calves. (**D**) Vaccinated-challenged calf showing no clinical signs, appearing clinically healthy.

**Figure 5:**
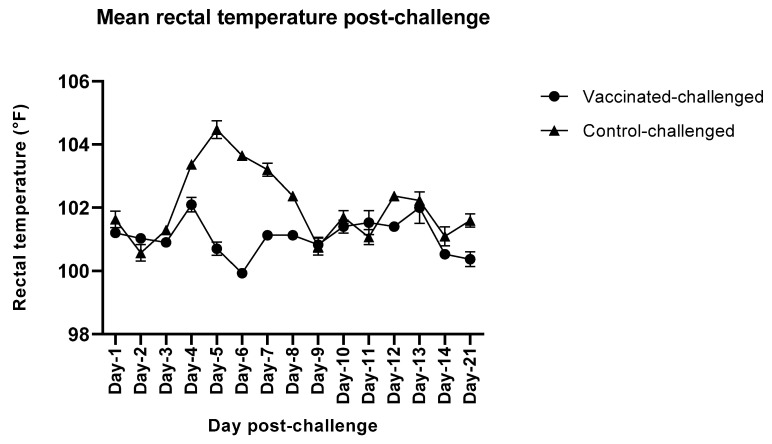
Mean rectal temperatures (±standard error) of vaccinated and control calves following experimental challenge infection with BVDV-1 virus.

**Figure 6:**
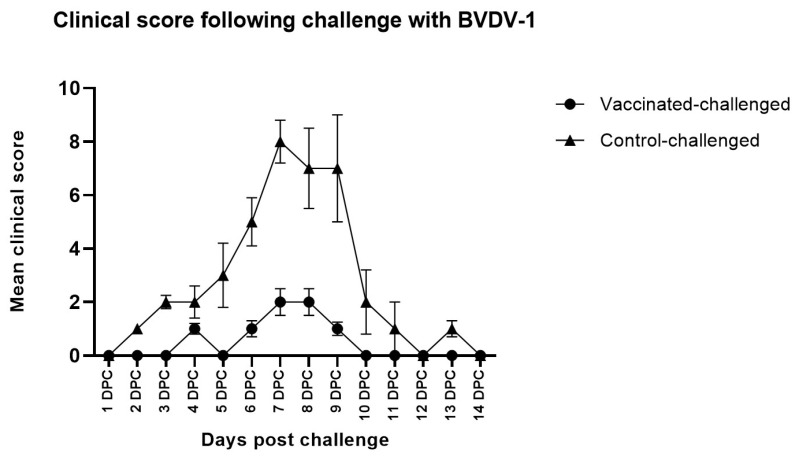
Assessment of clinical signs in calves following challenge infection with BVDV-1. Mean clinical score (±standard error) of calves following challenge infection with BVDV-1 in vaccinated and control calves.

The G2 and G4 calves were challenged with 5 mL of homologous live BVDV-1 virus (10^6.0^ TCID_50_ per ml) on 28 days post-booster (56th day of primary vaccination dose). All the control-challenged animals (G4) showed clinical signs like ocular discharge, nasal discharge and diarrhea following experimental BVDV-1 infection (Figure 4). All the control-challenged calves had fever (≥103 °F) with a mean duration of 3.8 days whereas only one out of three G2 calves (vaccinated-challenged) had fever on only one day (4 DPC) and the remaining two appeared healthy during the study period. Mean post-challenge rectal temperatures were significantly higher (*p* ≤ 0.01) in control calves as compared to the vaccinated calves (Figure 5). All control animals exhibited higher mean clinical score following challenge with BVDV-1 compared to vaccinates and the difference was significantly higher between 5 and 9 DPC (*p* < 0.002) (Figure 6).

#### 3.6.2. Virus Excretion in Nasal Secretions and Detection of Viraemia

BVDV RNA was undetectable in peripheral blood leukocyte (PBL) and nasal-swab specimens obtained from calves in groups G2 and G3 after both the primary and booster administrations of the inactivated vaccine, confirming the absence of viraemia and virus shedding through nasal secretions in vaccinated animals.

**Figure 7:**
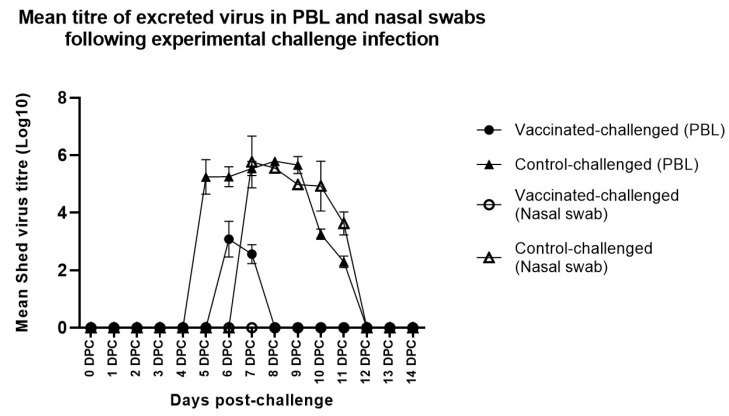
Detection of virus shedding by RT-qPCR from PBL and nasal swab samples of vaccinated-challenged and control-challenged calves (mean Log_10_ copy number/mL ± one standard error).

Following homologous BVDV-1 virus challenge, every calf in the control-challenged group (G4) showed detectable levels of viral RNA in PBL beginning on 5 days post-challenge (DPC) (mean = 1.78 × 10^4^ copies/mL), reaching a peak on 8 DPC (mean = 6.33 × 10^5^ copies/mL), and became negative by 9 DPC in one calf and by 11 DPC in all animals. In case of nasal swabs from the same group of animals, viral RNA first appeared on 7 DPC (mean titre = 5.9 × 10^5^ copies/mL), peaked on 8 DPC (mean titre = 3.6 × 10^6^ copies/mL), and was undetectable in one calf by 11 DPC and in the remaining calves by 12 DPC (Figure 7).

Among vaccinated-challenged calves (G2), viral RNA was detected in 1 of 3 animals’ PBL on day 6 and day 7 DPC (mean titre = 1.2 × 10^3^ and 3.6 × 10^2^ copies/mL respectively), whereas all nasal-swab samples remained RNA-negative throughout the 14-day observation period. These results demonstrated that the duration of viremia in the control-challenged group averaged 5.6 days, compared to only 0.66 days in the vaccinated-challenged group. Similarly, viral shedding via nasal secretions was observed for an average of 6.3 days in the control-challenged group, while no nasal shedding was detected in the vaccinated group. These findings indicate that BVDV-1 vaccination effectively prevented both viremia and nasal viral shedding.

#### 3.6.3. Virus Isolation from PBL and Nasal Swabs

**Figure 8:**
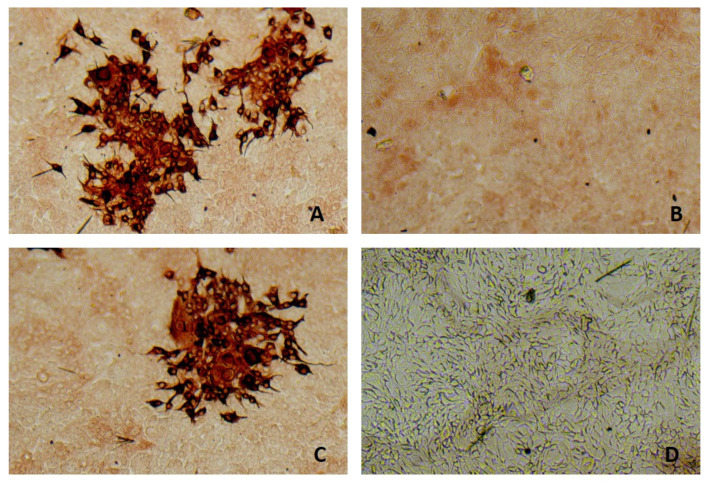
Virus isolation and detection by immunoperoxidase monolayer assay (IPMA) on MDBK cells. (**A**) Nasal swab sample from a control-challenged calf showing positive virus isolation. **(B**) Nasal swab sample from a vaccinated-challenged calf showing no virus isolation. (**C**) Peripheral blood leukocyte (PBL) sample from a control-challenged calf showing positive virus isolation. (**D**) Uninfected cell control.

Virus isolation results of PBL and nasal-swab samples from the challenged groups (G2, G4) showed that none of the three G2 calves that were RNA-positive in PBL on 6 and 7 DPC were positive by virus isolation. In the control-challenged group (G4), most of the RNA-positive PBL or nasal-swab samples yielded infectious virus; additionally, PBL from one calf was virus isolation-positive on 11 DPC despite being RNA-negative by real-time RT-PCR (Figure 8).

#### 3.6.4. IFN-Gamma Response Following BVDV-1 Vaccination

**Figure 9:**
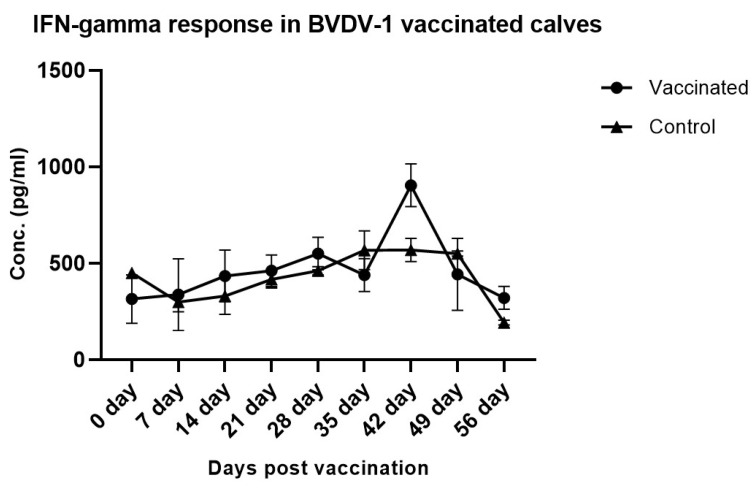
Temporal profile of serum IFN-γ concentration in BVD-vaccinated and control calves. Serum IFN-γ was measured from day 0 to day 63 post-vaccination. Vaccinated calves showed a distinct increase in IFN-γ, reaching a maximum at day 42, whereas control calves showed comparatively lower and fluctuating values over the same period.

Serum IFN-γ concentration was estimated from day 0 to day 56 post-vaccination in vaccinated and control calves to evaluate the cell-mediated immune response. The vaccinated group exhibited a progressive rise in IFN-γ levels up to day 28, followed by a sharp increase on day 42, when the peak response was recorded (Figure 9). Thereafter, IFN-γ concentration declined gradually through day 56. The control group showed comparatively lower and fluctuating IFN-γ values throughout the study period, with a marked decline toward the end of the observation period. These findings indicated that BVD vaccination elicited a distinct cell-mediated immune response in calves, as evidenced by increased serum IFN-γ concentration.

### 3.7. Safety and Immunogenicity in Field Cattle

**Figure 10:**
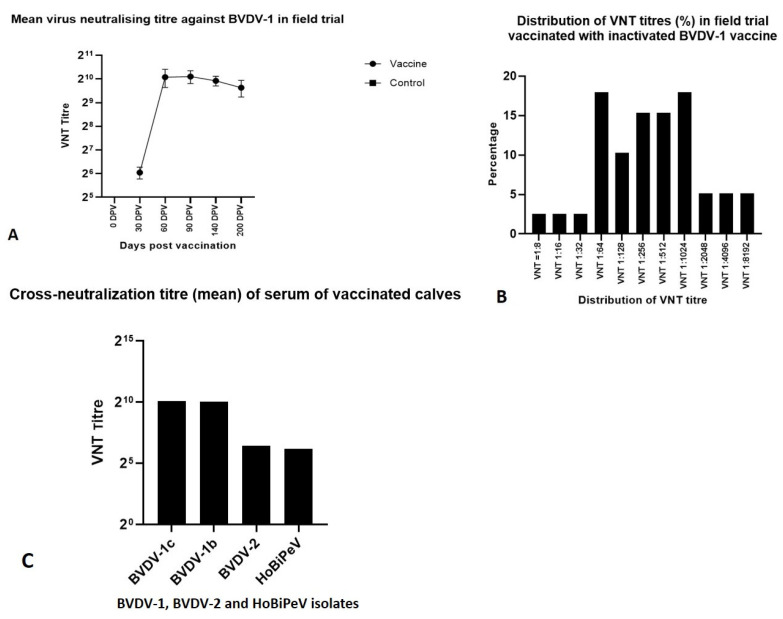
(**A**) Kinetics of serum immune response (VNT titres in log_2_ units) in BVDV-1 vaccinated cattle calves under a field trial. Control animals had negative (zero) VNT titres and were therefore not included on the logarithmic axis. (**B**) Distribution of VNT titres in BVDV-1 vaccinated calves under a field trial. (**C**) Mean cross-neutralization antibody titres of sera from BVDV-1 vaccinated calves against heterologous bovine pestiviruses (BVDV-1b, BVDV-2 and HoBiPeV).

A field trial was conducted to evaluate the immunogenicity of the vaccine in 125 cattle, comprising 100 vaccinated animals and 25 unvaccinated controls. Of the 125 enrolled animals, complete blood samples at all scheduled time points were obtained from 116 animals, including 96 vaccinated and 20 unvaccinated control cattle. Data from these animals were included in the final analysis. The remaining nine animals were excluded due to missed sample collections or because they could not be traced at one or more scheduled sampling time points during the study period. The vaccinated animals did not develop any adverse reactions during the study period. Among the vaccinated group, 74% achieved protective virus-neutralizing (VN) antibody titres of ≥7 log_2_ (≥1/128), 48% reached fetal protective titres of ≥9 log_2_ (≥1/512), and 92% maintained titres of ≥6 log_2_ (≥1/64) up to six months post-booster. The highest VN titre observed was ≥13 log_2_ (1/8192), and the mean titre at 90 days post-vaccination (two months after the booster dose) was 10.1 log_2_ (1/1100) in animals receiving a 5 mL dose of vaccine. None of the control animals developed detectable BVDV-neutralizing antibodies during the six-month study period. Cross-neutralization of heterologous bovine pestiviruses with serum from vaccinated animals revealed equivalent cross-neutralization titre against BVDV-1b and BVDV-1c strains, whereas a 3.5–4 fold reduction in neutralizing titre was observed against BVDV-2 and HoBiPeV (Figure 10).

### 3.8. Comparison of Immunogenicity Kinetics Between Guinea Pigs and Experimental Cattle

**Figure 11:**
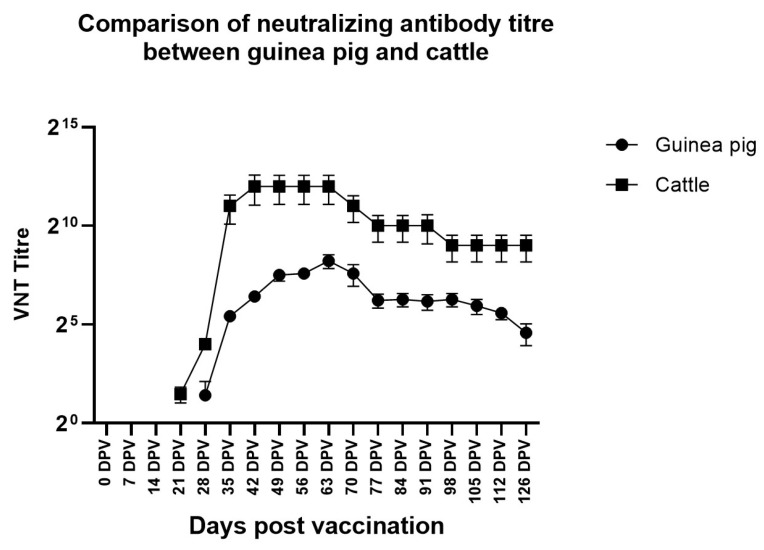
Comparison of kinetics of serum immune response (VNT titre in log_2_ units) in guinea pigs and cattle following immunization with inactivated BVDV-1 vaccine.

In calves, BVDV-specific antibodies began to appear by 21 days post-vaccination (DPV), which was one week prior to the administration of the booster dose. In contrast, in guinea pigs, antibody production commenced approximately one week after the booster dose (Figure 11). Additionally, peak antibody titres were observed at least three weeks earlier in calves compared to guinea pigs. Despite these temporal differences, the overall antibody responses in both species demonstrated a strong positive correlation, with a Pearson correlation coefficient of r = 0.8252, R^2^ = 0.6809, and a 95% confidence interval of 0.5829 to 0.9327 (*p* < 0.0001). A comparison of the immune responses in guinea pigs and cattle revealed that while both species exhibited similar kinetic patterns, the NA antibody titre levels were consistently higher in cattle. Additionally, the results showed that an NA titre of >5 log_2_ (>1/32) persisted from 35 to 112 dpv in guinea pigs indicating that >5 log_2_ (>1/32) NA titre at 6 weeks of primary vaccination (21 days post-booster) can be used as a criterion for evaluating immunogenic mass of a test BVD vaccine. These results suggest that guinea pig may be an alternative laboratory animal model to assess BVD vaccine safety and immunogenicity during batch testing.

### 3.9. Stability

The emulsion retained at 4 °C for 8 months exhibited antibody titre kinetics comparable to freshly prepared samples, with titres reaching up to 9 log_2_ (1/512) in guinea pigs. These results confirm the stability of the emulsion over the 8-month storage duration.

## 4. Discussion

Vaccination is an important measure with respect to both BVD control and limiting production losses associated with BVD. Although commercial inactivated BVD vaccines are available abroad, their protective efficacy against local circulating strains in India is uncertain due to the antigenic differences. Moreover, due to the marked genetic and antigenic variability among bovine pestivirus strains, WOAH has advocated for the development and use of BVD vaccines based on the predominant bovine pestivirus strains circulating in a country. As the current BVD vaccine was developed based on the genetic and antigenic data of bovine pestivirus strains circulating in India over the past 20 years, it offers not only wider clinical protection against BVDV-1 genotypes but also cross-protection against strains of BVDV-2 and HoBiPeV circulating in India. Apart from this, there is an added advantage of the field applicability of this vaccine, as promising results with regard to vaccine safety and immunogenicity were demonstrated in cattle under field settings. In this context, an effective indigenously developed inactivated BVD vaccine may provide a safer and potentially more efficacious alternative for regional disease control.

The present study comprehensively characterizes an inactivated BVDV-1 vaccine derived from an Indian field isolate and formulated with a water-in-oil adjuvant. The results of this study showed that the vaccine is safe, immunogenic and effective in protecting cattle against challenge-infection with BVDV-1, along with a reasonable cross-protective immune response against heterologous BVDV-2 and HoBiPeV. We used binary ethylenimine (BEI) as a virus inactivation agent and Montanide ISA 61 VG as an adjuvant for the preparation of the vaccine. In our formulation, complete inactivation was achieved using 3 mM BEI for 24 h at 37 °C, consistent with standard protocols for enveloped RNA viruses like foot-and-mouth disease virus (FMDV) and Newcastle disease virus (NDV) [23]. The use of binary ethylenimine (BEI) for virus inactivation has long been recognized as a superior method for maintaining the integrity of conformational viral epitopes [24,25]. The BEI inactivated vaccine formulation used in this study enabled robust induction of virus-neutralizing antibodies (VNAs), as evidenced by early seroconversion ≥3 log_2_ (1/8) titre by 28 DPV and sustained high titres up to 14 log_2_ (1/16,384) post-challenge, with ≥7 log_2_ (≥1/128) maintained for 12 months in vaccinated calves, thereby surpassing widely accepted protective VNT titre thresholds and performing comparably to, or exceeding, previous inactivated or modified-live vaccine platforms [10,26,27].

Montanide ISA 61 VG, a water-in-oil emulsion adjuvant, is known to induce a sustained release of antigen, favoring a depot effect that promotes prolonged antigen presentation and enhances humoral and cellular immunity [28]. Moreover, Montanide ISA 61 VG has been used in multiple veterinary vaccines and is particularly effective in ruminants, where it promotes a Th1/Th2-balanced response [29]. Also, the absence of residual infectivity or adverse systemic effects post-vaccination supports the formulation’s integrity and safety as observed in contemporary field and challenge studies [26,27,30]. Hence, Montanide ISA 61 VG was selected as the suitable adjuvant for the experimental vaccine. The ISA 61 VG formulated BVDV-1 vaccine in this study showed an early seroconversion (all ≥1/8 by 28 DPV) with peak titres reaching 1/16,384 post-challenge in vaccinated animals, indicating a robust protective power of this adjuvant.

After a homologous BVDV-1 challenge, a distinct dichotomy in clinical scores was observed. All control (unvaccinated-challenged) calves (G4) developed fever (≥103 °F, mean duration 3.8 days) and classical clinical signs (ocular/nasal discharge, diarrhea) whereas in vaccinated-challenged calves (G2), only 1 of 3 exhibited fever and only for a single day at 4 DPC. Importantly, vaccinated animals did not display respiratory or gastrointestinal symptoms, and the mean post-challenge rectal temperature was significantly lower in vaccinated versus control calves (*p* ≤ 0.01), underscoring a robust protective effect. Studies on both inactivated and modified-live BVDV vaccines consistently demonstrate significant reductions in post-challenge clinical signs, including fever and mucosal or enteric symptoms, which are considered important efficacy endpoints in addition to the primary criterion of fetal protection for vaccine licensure and field use [31,32,33,34]. The magnitude of reduction in clinical scores seen here is comparable to that reported earlier, where vaccinated calves exhibit either subclinical or markedly attenuated clinical disease versus unvaccinated controls [32,33].

The virus-neutralizing antibody (VNA) titre profile in calves vaccinated with the inactivated BVDV-1 vaccine candidate is an indicator of effective humoral immune induction, durability of protection, and likely field relevance. In this study, detectable neutralizing antibodies appeared as early as 21 days post-vaccination (DPV), with initial titres (up to 1/16 in some animals) signaling efficient priming. By 28 DPV, all vaccinated calves seroconverted, reaching titres between 3 and 6 log_2_ (1/8 and 1/64). The magnitude of the antibody response peaked by 56 DPV (day of challenge), where mean titres reached 12.2 log_2_ (approximately 1/4778.7), with the highest individuals at 13 log_2_ (1/8192). This is consistent with an earlier study which showed that neutralizing antibody responses developed earlier and to higher concentrations in calves after infection with noncytopathic BVDV [35]. There is no scientific consensus yet to clearly define a threshold of NA at which animals would be protected against BVDV infection. Although a previous study showed that a VNA titre of 9 log_2_ (1/512) was necessary for marked protection against BVDV infection in cattle [36], protective effects appeared with a titre of 8 log_2_ (1/256) [37]. In contrast, another study showed that the nAb titre of 2.4 log_2_ and above is sufficient to prevent clinical signs of BVDV infection and viremia [10]. Therefore, the VNA titre of vaccinated calves in this study substantially exceeds the widely accepted NA protective threshold of 7 log_2_ (1/128) for prevention of clinical disease, as well as 9 log_2_ (1/512), the titre reported to be adequate for fetal protection during gestation [26,38]. Such results underscore both the robust immunogenicity of the vaccine and the capacity for strong memory B-cell responses, particularly with adjuvanted inactivated formulations [26,27,39].

Another important finding of this study is the persistence of high homologous BVDV-1 NA titres for at least a year: mean titres of 9 log_2_ (1/512) were maintained for five months, 8 log_2_ (1/256) for nine months, and 7 log_2_ (1/128) for twelve months in vaccinated-unchallenged animals, indicating a stable NA response for at least 1 year induced by the vaccine. This stable and prolonged maintenance of NA response is very important, supporting the strategic aim of annual vaccination in cattle management. The duration of vaccinal immune response compares favorably with both field and experimental data, where faster waning after inactivated vaccines often necessitates more frequent boosters [26,34,40,41]. The persistence of fetal-protective VNA titres ≥ 9 log_2_ (≥1/512) for up to five months is of particular importance in preventing vertical transmission and generation of persistently infected (PI) calves, one of the key drivers of BVDV epidemiology [38,42].

In a previous study, the average homologous neutralizing antibody titre elicited by inactivated BVDV-1a vaccines was reported to be 7.1 log_2_ (1/140), while the response against BVDV-1b was slightly lower, averaging 6.6 log_2_ (1/96) [43]. In this study, the maximum average neutralizing antibody titre detected against the homologous virus (BVDV-1c) was 12.2 log_2_ (1/4778), which is significantly higher than those reported in prior studies. Moreover, the neutralizing antibody titre against the BVDV-1b strain was equivalent, indicating robust cross-protective immunity against the more prevalent BVDV-1b genotype. Fulton et al. (2020) [44] also observed similar neutralizing titres ranging from 2 to 10 log_2_ (1/4 to 1/1024) between BVDV-1a and BVDV-1b following vaccination with a killed BVDV-1 vaccine. In a high-dose vaccination study (10^7.8^ TCID_50_) conducted by Beer et al. (2000) [36], mean titres of 10.72 and 11.38 log_2_ (1/1687 and 1/2679) were observed against two different BVDV strains, with a reported range of 9 to 13 log_2_ (1/512 to 1/8192), representing the highest antibody levels achieved with a killed vaccine in calves to date. Notably, in our study, similar titres were achieved using a lower antigenic dose (10^6.0^ TCID_50_/mL).

Following live virus challenge, vaccinated calves exhibited a pronounced anamnestic response, with peak titres rising further to 14 log_2_ (1/16,384) at 84 DPV, whereas control animals only began to seroconvert two weeks post-challenge and demonstrated delayed, lower-magnitude responses. This robust secondary response in vaccinates highlights their primed cell populations and effective B-cell memory [27,41], mirroring outcomes seen with highly effective live and vectored vaccines [32,45,46].

This study demonstrated that BVDV-1 vaccination elicited a detectable cell-mediated immune response in calves, as evidenced by changes in serum IFN-γ concentration over the post-vaccination period. A gradual increase was observed in the vaccinated group, with a marked peak around day 42, followed by a decline toward the later sampling points. This pattern is suggestive of antigen-driven T-cell activation and the development of adaptive immune memory. In contrast, the control group showed only minor fluctuations without a comparable sustained increase, indicating that the response observed in vaccinated calves was specifically associated with immunization. Since IFN-γ is a major Th1 cytokine involved in antiviral defense, its elevation supports the ability of the vaccine to stimulate the cellular arm of immunity, which is essential for effective protection against BVDV.

Studies on field trials have demonstrated that inactivated BVDV vaccines elicit neutralizing antibody titres above 7 log_2_ (1/128) in a majority of well-primed animals, yet maintenance of titres above 9 log_2_ (1/512), a hallmark of reliable fetal protection, is limited. The NA titres observed here, achieved and sustained without evidence of adverse reactivity and bolstered by recall ability post-exposure, suggest superior or at least comparable potency to best-in-class inactivated vaccines [27,45]. This stands in contrast to certain commercial inactivated vaccines, which may struggle to generate durable, high-magnitude titres in the field, especially following a single dose [40].

Experimental studies with inactivated BVDV vaccines have demonstrated that vaccination against BVDV-1 can confer protection against BVDV-2 [33,36,47]. Nevertheless, field observations indicate that vaccinated animals may still become infected with heterologous strains [48,49]. A serum neutralization titre of 4 log_2_ (1/16) has been suggested as the minimum threshold for cross-protection between BVDV-1 and BVDV-2 [50]. In this study, results of cross-neutralization tests of BVDV-1-vaccinated sera with heterologous BVDV-1 strain (BVDV-1b) found no significant differences in NA response against BVDV-1c and BVDV-1b, indicating a robust immunity against locally circulating BVDV-1 strains in India. Additionally, cross-neutralization assays conducted with BVDV-1 vaccinated experimental and field cattle sera and strains of heterologous bovine pestivirus species (BVDV-2, HoBi-like virus) revealed a moderate level of cross-protective immunity, which is encouraging given the documented antigenic divergence within bovine pestivirus species circulating in India [5,7,15,16]. Our findings also align with prior observations that whole-virus vaccines can induce partial heterologous immunity [47,51].

Another important finding of this study is the reduction in virus replication and shedding in vaccinated animals following the homologous BVDV-1 virus challenge. All calves in group G4 exhibited systemic viral replication, evidenced by the early detection of viral RNA in PBL at 5 days post-challenge (DPC) (mean = 1.78 × 10^4^ copies/mL), which is consistent with the onset of acute viremia observed in experimental BVDV infections [52]. This systemic phase peaked by 8 DPC (mean = 6.33 × 10^5^ copies/mL) before a rapid decline, with viral RNA becoming undetectable in PBL by 11 DPC in all animals. This general profile parallels prior reports on acute infection in seronegative calves [52,53]. Whereas, in vaccinated-challenged animals (G3), viraemia was absent in two animals, and was limited to only two days in one vaccinated animal. In this study, local viral replication in the upper respiratory tract was indicated by RNA detection in nasal swabs starting at 7 DPC (mean titre = 5.9 × 10^5^ copies/mL), reaching its maximum at 8 DPC (mean = 3.6 × 10^6^ copies/mL), and declining to undetectable levels by 12 DPC. The lag in detection of nasal viral RNA compared to PBL likely reflects initial systemic dissemination followed by local replication and shedding through the respiratory mucosa [54]. Importantly, the virological course observed in group G4 mirrors natural acute BVDV infections. A reduced or absent viremia and nasal viral shedding in vaccinated calves is a hallmark of effective BVDV vaccines [33,55].

The field cattle trial under natural settings confirmed the vaccine’s real-world applicability as evidenced by optimal immunogenicity and no adverse clinical findings. A 92.8% retention rate (96/100 vaccinated, 20/25 controls) for serial sampling strengthens the validity of these findings, limiting bias from attrition and ensuring adequate statistical power. Within the vaccinated cohort, 74% attained virus-neutralizing (VN) titres ≥7 log_2_ (≥1/128), a threshold widely accepted as conferring clinical protection against acute BVDV and suppressing horizontal transmission [33,42,56]. This rate compares favorably to established efficacy criteria, often 70% or higher, achieving protective titres for licensure and deployment in herd-level BVDV control strategies [34,42]. Also, 48% of vaccinated cattle in field settings achieved the higher threshold of ≥9 log_2_ (≥1/512), which correlates with fetal protection. Attaining and maintaining such titres is linked with prevention of transplacental BVDV infection and, consequently, a reduced risk of fetal loss or generation of persistently infected (PI) calves [56]. Sustained VN titres of ≥6 log_2_ (≥1/64) in 92% of vaccinated animals up to six months post-booster in this study indicate a better durability of humoral immunity. While ≥6 log_2_ (≥1/64) is viewed as the minimal threshold for protection [34], maintaining this in the vast majority of animals over half a year aligns with annual (or even biannual) vaccination schedules, facilitating integration with typical cattle management and breeding cycles.

Guinea pigs are established experimental models for testing the immunogenicity and efficacy of bovine vaccines against bovine alphaherpesvirus 1 (BoAHV-1) [57,58,59]. Moreover, guinea pigs are increasingly being recognized as a suitable surrogate model for evaluating BVDV vaccine immunogenicity, owing to their dose-dependent neutralizing antibody responses that correlate well with those observed in cattle [60]. A previous study compared the humoral response of the BVD vaccine in cattle and guinea pigs and showed a correlation between them [61]. In contrast, another study [62] evaluated the serologic response of bovines and guinea pigs immunized with an inactivated vaccine and showed that the response was inconsistent and of low magnitude. However, another study [63], assessing serological response to BVDV-1 vaccines, showed production of neutralizing antibodies in vaccinated guinea pigs, with most vaccinated animals seroconverted after the second administration, indicating the utility of guinea pigs to assess BVD vaccine immunogenicity. 

In this study, seroconversion in guinea pigs began with modest neutralizing antibody titres (1/8) in a subset of animals by day 28, but full seroconversion in all immunized animals was observed by day 35 post-vaccination (DPV), which is one week earlier than previously reported (42 DPV) [63]. Notably, although high antibody titre was found in vaccinated cattle, the overall antibody responses between cattle and guinea pigs in this study demonstrated a strong positive correlation (r = 0.8252, R^2^ = 0.6809, *p* < 0.0001). This finding is consistent with an earlier study on bovine-guinea pig comparisons (R^2^ = 0.71, *p* < 0.0002) upon inactivated BVDV vaccination [39]. The findings in our study demonstrate that the antibody levels in vaccinated guinea pigs can be used as a reliable tool to predict BVDV-1 vaccine immunogenicity in cattle, as cattle trials are complex, costly, and time-consuming, particularly in countries where cattle slaughter is prohibited. Although BVDV-1 is specific to cattle and other ruminants, guinea pigs can be used to predict how the immune system in cattle will respond to BVDV-1 vaccination.

However, this study has a few limitations such as the vaccine efficacy challenge study in experimental cattle, which was against the homologous BVDV-1 virus, and a limited number of animals were used per group in the challenge experiments. Hence, expanded efficacy trials with larger cohorts and in pregnant cattle may be required.

This study shows great promise for preventive vaccination of cattle against BVD, since the vaccine formulation prepared using a local BVDV-1 strain and water-in-oil-based adjuvant protected vaccinates against homologous virus and enabled the development of moderate cross-protective antibody responses against heterologous BVDV-2 and HoBiPeV. Additionally, the duration of the protective immune response in vaccinated cattle lasted for a year.

## 5. Conclusions

In conclusion, this study demonstrated that the inactivated BVD vaccine candidate developed using a local Indian BVDV-1 strain and Montanide ISA 61 VG adjuvant is safe, highly immunogenic, and confers robust protection against homologous virus challenge in cattle. Notably, this vaccine provided a sustained protective immune response against the homologous virus which lasted for a year, with a reasonable cross-protective humoral immune response against heterologous BVDV-2 and HoBiPeV strains. Additionally, our results demonstrated a strong positive correlation in neutralizing antibody levels between cattle and guinea pigs, suggesting that guinea pigs can be used as a reliable experimental animal model to predict BVDV-1 vaccine immunogenicity in cattle. The performance of the vaccine candidate under both controlled and field conditions provides a strong foundation for further evaluation and its eventual use in the field for prevention and control of BVD in India.

## Data Availability

All required data are available as texts and figures in main text of the article.
